# Critical Review: The Past, Present, and Future of Small Intestinal Bacterial Overgrowth (SIBO)

**DOI:** 10.1002/jgh3.70419

**Published:** 2026-05-15

**Authors:** Ayesha Shah, Gerald Holtmann, Peter R. Gibson

**Affiliations:** ^1^ Faculty of Health, Medicine and Behavioural Sciences The University of Queensland Brisbane Queensland Australia; ^2^ Department of Gastroenterology & Hepatology Princess Alexandra Hospital Brisbane Queensland Australia; ^3^ Department of Gastroenterology School of Translational Medicine, Monash University Melbourne Victoria Australia

## Abstract

Small intestinal bacterial overgrowth (SIBO) has evolved from a severe debilitating illness to a clinical spectrum associated with abnormalities of microbial populations in the small intestine. It is best described as a pathophysiological state that may contribute to symptoms or other pathology in a wide range of gastrointestinal conditions that include disorders of gut‐brain interaction, inflammatory bowel disease, and chronic liver disease. The controversy surrounding SIBO largely rests on diagnostic limitations of currently widely used diagnostic tests. The gold standard test of bacterial culture of proximal small intestinal fluid will miss the majority of bacteria, has severe methodological limitations, and is clinically uncommonly used. Breath‐hydrogen testing after oral glucose or lactulose ingestion is associated with falsely positive and negative results, due largely to the inability to accurately define where in the gastrointestinal tract the hydrogen is being generated. Such performance characteristics raise concerns regarding its application to clinical decision‐making in the individual patient. Therapies such as antibiotics can normalize breath test findings and ameliorate symptoms, but whether this is due to an action on the small intestinal microbiota is uncertain. It is proposed that conceptual modifications that include the assessment of the mucosa‐associated microbiota in biopsies obtained with minimal contamination, molecular and functional characterization of the microbiota, rather than purely culture‐based density of bacteria, and expansion of the definition of SIBO to small intestinal dysbiosis will facilitate more precision and resolve the controversial place of SIBO in clinical practice.

## 1 Introduction

Small intestinal bacterial overgrowth (SIBO) is one area of gastroenterology that is steeped in controversy. The concept that the small bowel microbiota might be a contributor to the genesis of symptoms and illness, especially in chronic intestinal disorders such as disorders of gut‐brain interaction (DGBI) or in chronic liver diseases, is attractive as it provides mechanistic bases for observations and is potentially amenable to therapy with improved outcomes. The controversy arises not from the concept of SIBO itself, but from the fact that an extensive literature is largely based upon imprecision in diagnosis and subsequent therapeutic trials and the lack of specific, validated, and universally accepted diagnostic and therapeutic interventions for SIBO. Disagreement and controversy have emerged as a consequence of how diagnostic tests and the therapeutic approach currently recommended by some guidelines should be applied in routine clinical practice. This review aims to address the reasons behind the confusion and disagreement with a view to how greater precision might resolve the controversy.

## 2 The Origin of the Concept of SIBO

The first recognition of SIBO has been credited to Faber, who described a patient with “blind loop syndrome” in 1897, with several subsequent case reports and a detailed description of the pathophysiology and therapy of the blind‐loop syndrome by Booth and Mollen in 1960 [1]. SIBO was a syndrome of ill health that might include weight loss, diarrhea, maldigestion and malabsorption and that was causally associated with an increased microbial load in the small intestine. It was observed in the setting of stagnation of small intestinal contents due to abnormal anatomy like intestinal strictures, surgically created blind loops (often associated with peptic ulcer surgery), jejunal diverticula or gross hypomotility associated especially with severe scleroderma. Pathogenic mechanisms by which a high density of luminal bacteria in the small bowel might disturb small intestinal homeostasis and function and lead to the clinical scenario were multiple (Table 1). The diagnosis was relatively easily made by demonstrating the anatomy of the upper small intestine combined with bacterial culture of jejunal aspirates that showed > 10^5^ cfu/mL. New noninvasive tests were developed, such as the ^14^C‐bile acid breath test, which showed early bile acid deconjugation after oral ingestion of a conjugated bile acid, and breath hydrogen tests, from which a double peak and/or early rise of breath hydrogen after ingestion of lactulose indicated fermentation of the lactulose in the small bowel. Therapy comprised nutritional support, antibiotics and, most critically, correction of the anatomical abnormality if possible.

**TABLE 1:** Pathogenic mechanisms associated with classical SIBO.

Key physiological structure/functions essential for small intestinal homeostasis and function	Disturbances in SIBO
Flowing/flushing system → discourages luminal growth of bacteria adherent to fiber and other particles	Stasis due to anatomical abnormality or gross hypomotility counteracts flushing mechanism
Large surface area to absorb nutrients due to villi	Villous blunting related to mucosal inflammation
Mucus is single‐layered without the colonic mucous structure of double‐layering with sterile inner layer	Increased bacterial density → increased proximity of bacteria to epithelium → mucosal inflammatory response to bacteria
Aerobic environment Facultative anaerobes will metabolize sugars via respiration, not fermentation Strict anaerobes not likely to be active (e.g., methanogens [archaea], sulphate‐reducing bacteria)	Sump of bacteria → consumption of oxygen → anaerobic → switch of facultative bacteria to carbohydrate fermentation for energy → metabolites such as SCFA, hydrogen, H_2_S → epithelial injury
Epithelium Greater permeability than in the colon Major energy source is luminal glutamine Ability to absorb SCFA^a^ < 50% of that in colon—not built to handle lots of SCFA from bacterial fermentation	Mucosal inflammation and epithelial injury → ↑paracellular permeability Luminal glutamine consumption of bacteria → ↓availability SCFA exposure greater than capacity to oxidize for energy
Luminal factors to assist in digestion and absorption; e.g., luminal and surface‐associated enzymes; conjugated bile acids → micellar formation	Deconjugation of bile acids → failure of micellar formation → maldigestion of fate, malabsorption of fat‐soluble vitamins

^a^ SCFA, short‐chain fatty acids.

## 3 Expansion of the Concept to a SIBO Spectrum

In the late 20th century, the concept was expanded on the idea that the “classical” SIBO was the severe end of a spectrum and that expanded bacterial populations in the small intestine might cause symptoms in the absence of gross changes to the structure or function of the intestinal mucosa. At the mild end of this spectrum, stasis of intestinal contents was not a necessary condition for its presence. Breath hydrogen tests were used to identify excessive fermentative activity within the small intestine and cut‐off values for bacterial counts in small bowel aspirates were reduced from 10^5^ to 10^3^ cfu/mL. Additional pathogenic mechanisms were incited and mainly involved the microbes detected in the aspirates and/or the metabolites they produced. Each gained its own acronym. Thus, small intestinal fungal overgrowth (SIFO) based on identifying fungi in small bowel aspirates in symptomatic patients was described [2], although their pathogenic involvement with symptom genesis has yet to be shown. Intestinal methanogen overgrowth (IMO) based upon concentrations of methane in the breath and intestinal sulfide overproduction (ISO) based upon sulfides putatively detected on the breath have been proposed [3], but their actual production in the small intestine, as opposed to phenotypic presence [4], has yet to be shown. With improved phenotypic recognition of microbiota, the concept that it is not only the density of luminal bacteria, but also the composition diversity and more importantly the metabolic function of the bacteria, and the potential involvement of the mucosa‐associated microbiota that are relevant in the pathophysiology.

## 4 Diagnostic Testing

The whole narrative around the importance of SIBO centered on its recognition. In clinical practice, two main approaches have been used to detect SIBO—culture of small intestinal fluid and breath hydrogen test after glucose or lactulose.

### 4.1 Culture of Small Intestinal Fluid

While taking a duodenal or jejunal aspirate at upper gastrointestinal endoscopy for culture would seem a simple thing to do, few centers do this routinely. The reasons for the poor uptake can be attributed to several factors [5, 6]. First, obtaining fluid deep in the duodenum is not easy, and may require, for example, introduction of saline via the scope to enable its capture, an addition that surely would cast doubt upon the meaning of the bacterial counts expressed per mL. Second, the use of air insufflation during the procedure may influence the microbial yield, particularly of anaerobes. Third, obtaining an aspirate that is not contaminated by oropharyngeal secretions or other fluids is essential. Specialized catheters that minimize contamination have been subsequently developed [5, 7]. Fourth, the use of culture to identify microbes is problematic given that a majority of microbes cannot be cultured. Their accurate quantification requires a dedicated microbiological approach not usually available in routine laboratories. Fifth, luminal microbiota is phenotypically and functionally different from that in the mucosa‐associated compartment [7, 8]. The relative importance for clinical manifestations and pathology of the microbiota from this compartment, which can be accessed by endoscopic biopsy, is only now being examined, as discussed later. Finally, the techniques for collection of fluid and the culture methodology have not been standardized, and this raises questions about interpretation of the results, particularly when compared across different centers. That bacterial counts from jejunal/duodenal aspirates comprise the “gold standard” for detecting increased density of small intestinal bacteria raises concerns of accuracy and precision.

### 4.2 Breath Hydrogen Testing

The induction of increase in breath hydrogen > 12–20 ppm over baseline level after an oral dose of lactulose or glucose in a fasting person indicates that the sugar is being fermented by bacteria [9]. Hydrogen is released with 5 min from bacteria after exposure and appears in the breath with a very short lag period [10]. Methane can be used as the readout gas in so‐called non‐hydrogen producers. Hydrogen and methane are only produced in the gastrointestinal tract. The big challenge of such methodology is how to recognize the gas is coming from the small intestine and not from the major fermentative part of the gut, the colon.

An oral dose of lactulose is exposed to the entire small bowel and proximal colon since it is not hydrolyzed by small intestinal disaccharidases and is not absorbed. Hence, it will be fermented in the colon raising the challenge of defining its small intestinal origin. A double peak of hydrogen, the first in the small bowel and second when the lactulose reaches the caecum, was considered diagnostic of SIBO, but double peaks can be observed when the lactulose reaches the caecum without SIBO [11]. Illogically, high fasting breath hydrogen and even very high concentrations of breath hydrogen have been used to reflect SIBO with no validation. On the assumption that orocaecal transit was no less than 90 min, it was hypothesized that a rise in breath hydrogen within 90 min of its ingestion would indicate the small bowel origin of the hydrogen. This assumption was shown to be erroneous by concurrent ingestion of ^99m^Tc sulfur‐colloid with lactulose; in the vast majority of tests, ^99m^Tc was in the caecum before the hydrogen rise and that only 0.5 g of lactulose is needed to cause that rise [11, 12, 13]. Hence, to enable specific detection of SIBO by the time of the hydrogen rise, concurrent scintigraphic assessment would appear mandatory [11].

An alternative is to use glucose as the ingested sugar. The assumption is that glucose is rapidly and completely absorbed in the proximal small intestine and, therefore, a rise in breath hydrogen must represent SIBO. High doses of 50 or 75 g are used to reduce false negatives, but at those doses, the assumption about glucose being completely absorbed is erroneous since high rates of glucose malabsorption may be observed [14, 15]. Hence, the test can theoretically be falsely positive and falsely negative.

When compared with jejunal culture as the gold standard in a pooled analysis, breath testing (without scintigraphy) with lactulose has a sensitivity of 42% and specificity of 71%, and with glucose, has a sensitivity of 55% and specificity of 83% [16]. The better performance of glucose over lactulose and the reduction of false positive tests have led to glucose breath test being the preferred clinical test for SIBO in clinical guidelines [9]. False‐negative studies are less likely with lactulose and the high false‐positive rate can be minimized by concurrent scintigraphy, but this combined breath and scintigraphic test is not commonly applied.

### 4.3 Breath Test Findings in Health and Disease States

Whether breath tests can distinguish healthy from SIBO has been evaluated across multiple chronic gastrointestinal and hepatological conditions. The power of meta‐analysis has been used as data from large populations are likely to reduce the noise from the imprecision associated with the tests. Abnormal breath tests are observed in the healthy controls but are more prevalent in, for example, IBS, functional dyspepsia, inflammatory bowel disease, celiac disease, and advanced chronic liver disease [17, 18, 19, 20]. The methodology from the original studies is heterogeneous, and the data are of low quality. While such analyses indicate abnormal breath tests and presumably SIBO are likely to be more prevalent in those conditions than in health, they provide limited information into, for example, risk factors and associations that might provide insight into pathogenic aspects of those conditions or who might benefit from antibiotics. Using the Bradford‐Hill criteria for causation [21], the higher prevalence of abnormal findings across multiple disease states raises concern that the findings are less likely to be causally associated.

## 5 Breath Test‐Directed Therapy

The main therapeutic options that have been subject to randomized controlled trials have included antibiotics (rifaximin, quinolones, aminoglycosides, norfloxacin, metronidazole, chlortetracycline and neomycin), prokinetic agents (cisapride, mosapride and berberine), probiotics of varying composition, and ursodeoxycholic acid, alone or in combination. Nearly all studies used lactulose and glucose breath tests, alone or in combination, to make the diagnosis and to assess eradication of abnormalities. For example, only one of 30 studies that were analyzed by network meta‐analysis [22] used jejunal culture for diagnosis (a study of nine patients only). There was heterogeneity in the diagnostic criteria used, which were beyond the scope of the current review. Efficacy of therapy can be looked at in two ways—eradication of the breath test abnormalities, the focus of most studies, and improvement in the clinical problem, such as abdominal symptoms in patients with DGBI.

### 5.1 Breath Test Normalization

In the latest published network analysis [22], most therapies for which sufficient studies had been performed to compare with placebo were more effective at normalizing the breath test findings. Thus, the relative risk (RR) for normalizing breath tests was, for example, 2.4 (95% CI: 1.3, 4.5) for rifaximin, 3.4 (2.3, 4.9) for probiotics, 7.5 (1.1, 49.5) for prokinetic agents and 8.4 (1.1–64.2) for aminoglycosides. The probability for being an ideal intervention (via the surface‐under‐the‐cumulative‐ranking‐curve [SUCRA] values) was calculated for each intervention in the network analysis to create an efficacy hierarchy. For example, the combination of rifaximin and a prokinetic drug (67%), quinolones (64%), aminoglycosides (59%), rifaximin as a monotherapy (58%), probiotics (55%), prokinetic agents (54%) and metronidazole (40%) performed well, but ursodeoxycholic acid (26%), tetracyclines (17%) and placebo (6%) did not [22]. In terms of actual eradication rates, meta‐analyses for rifaximin, for example, reported eradication to be highly variable, but averaged out per‐protocol at 73% (*n* = 1331 patients from 32 studies) [23] and 63% (*n* = 874 patients from 24 studies) [24]. A problem across all these studies was the high heterogeneity of diagnostic methods and criteria used to assess both SIBO and its eradication. Nevertheless, it seems well established that the majority of therapies reported can normalize the breath test results in the majority of patients.

### 5.2 Symptomatic Response to Breath Test Normalization

It is reasonable to believe that eradication of breath test abnormalities would predict symptomatic response to antibiotics. Such findings were reported for rifaximin (87% response with breath test normalization compared with 7% where abnormalities remained) [25] and for neomycin in patients with IBS where the responses were 62% compared with 34%, respectively, although placebo responses were seen in only 4% [26]. In a systematic review, only 10 studies enabled assessment of symptomatic response when SIBO was eradicated and this averaged at 68% [23]. On the other hand, several studies have not reported a relationship of breath test normalization with symptomatic improvement [27, 28, 29, 30]. The most recent was a randomized comparison of rifaximin with a low FODMAP diet in 100 patients with IBS. Both achieved a comparable efficacy at 4 weeks of about 50% [30]. Glucose breath test normalization was similar in each arm (50%–64%) and there was no association with symptomatic response. To confuse further, a retrospective analysis of a large cohort of patients with IBS treated with antibiotics identified a flat‐line breath test, but not signs believed to reflect SIBO, was the best predictor of symptomatic response [31].

## 6 The Controversy of the Current Status of SIBO in Clinical Practice

Faced with an individual patient, the clinician must decide whether the accuracy of breath testing is good enough to make a diagnosis in a symptomatic individual, to give the patient a label that is associated with considerable and often unsubstantiated advice by internet experts, and to make therapeutic decisions that often involve the use of antibiotics that may commit the patient to repetitive courses. The key issues underlying this controversy might be summarized in the following four areas:

- *Inaccuracy in diagnosis*: The accuracy of a diagnosis [16] implies that at least one in four patients do not have SIBO. Likewise, one in four who are considered not to have SIBO may have it.
- *The assessment of eradication is inaccurate*: Since the success of therapy is assessed using the same diagnostic test, inaccuracies will be compounded.
- *Symptoms are not specific for SIBO*: SIBO cannot be recognized by the clinical presentation. Successful eradication of breath‐test diagnosed SIBO may not, therefore, ameliorate the symptoms that may have been attributed to the SIBO.
- *Therapies for SIBO are not specific for the small intestine*. Antibiotics, prokinetic agents, and probiotics do not act specifically on the small intestine. For example, antibiotics used are often poorly absorbed. Hence, the drug will also enter the colon and have antibiotic effects there too, raising uncertainty as to whether its effects on the colonic or small intestinal luminal bacteria, or both, underly symptomatic improvement. The same scenario applies to the low FODMAP diet, which reduces the abundance of bacteria in the colon (as reflected in the feces [32]). Furthermore, reduced bacterial lipopolysaccharide load within the large bowel may be a mechanism by which FODMAP restriction might work in IBS [33]. Dietary reduction of FODMAPs probably also reduces the density of bacteria in the small intestine since it normalized glucose breath tests in about 50% of patients with IBS, similarly to rifaximin [30]. Hence, attributing symptomatic response with diet to a patient who has SIBO may be incorrect.

## 7 The Future

As alluded to above, current approaches to assessing the small intestinal microbiome can be described as “imprecision medicine,” with the limitations of the currently available tests for diagnosing SIBO being well recognized [16, 34]. In order to advance to contemporary “precision medicine,” better diagnostic tools are needed for which there has been broad recognition [35].

To characterize microbial colonization of the small intestine effectively, we propose that our framework must extend beyond the traditional SIBO concepts to recognize and understand the broader concept of microbial dysbiosis. Small intestinal dysbiosis is defined as the changes in composition, density, and function of small intestinal mucosa‐associated microbiota [36]. It is well established that small intestinal dysbiosis contributes to the pathogenesis of, and in some cases drives the progression of, intestinal and extraintestinal disorders [37].

However, historically, the small intestine was viewed as largely sterile or minimally colonized compared with the large intestine, due to gastric acid and high concentrations of digestive enzymes and bile [38]. Bacteria were thought to be present only from cross‐contamination or in the context of SIBO. Thus, until recently, most microbiome research focused on the stool microbiome because its relatively easy access and high microbial biomass make fecal samples an attractive resource for study [39]. However, fecal microbiota fails to capture the spatial heterogeneity of the microbes colonizing the gastrointestinal tract. They do not reflect longitudinal variation in microbial composition along the gastrointestinal tract, and the regional (anatomic) variation, where different intestinal compartments host distinct microbial communities. Finally, fecal microbiota is largely influenced by diet and intestinal transit [40]. Thus, the fecal microbiome does not represent microbes that are attached to and embedded within the mucus layer, in close proximity to the epithelial lining of the gastrointestinal tract, commonly referred to as the mucosa‐associated microbiome (MAM) [39].

On the other hand, the large surface area of the small intestine, including the mucosal folds, villi and the microvilli, along with the microbes colonizing the MAM play a crucial role in host physiological functions, such as the digestion of food, absorption of micronutrients, production of vitamins [41], maintenance of immune homeostasis [42] and intestinal barrier function [43]. Multiple recent studies have demonstrated small intestinal microbial dysbiosis via compositional alterations in microbial communities, principally via taxonomy‐based assessments using 16S ribosomal RNA (16S rRNA) gene amplicon sequencing [41], and more recently, shotgun metagenomic sequencing applied to samples of luminal bacteria [44] play an important role in various gastrointestinal conditions.

Culture‐independent omics approaches now reveal a much more complex gut ecosystem [45]. Metagenomics analyses the gene repertoire present in a microbial community and how it may be expressed under different conditions, while metatranscriptomics focuses on actively expressed genes and regulatory networks. When combined with metaproteomics and metabolomics, these techniques illuminate not only which microbes are present and what genes are expressed, but also the proteins produced and the metabolites generated, providing a functional view of microbial activity and its potential effects on the host. Together, these omics strategies address key questions about community composition, gene expression, and ecological function under varying conditions, and how microbial products influence the health of the gut environment.

While these approaches have shown how the taxonomic and functional diversity of the intestinal microbiota may influence human physiology in health and disease, several knowledge gaps remain. We highlight below cutting‐edge, fast‐evolving methods that could soon be integrated into routine clinical practice for characterizing small intestinal microbial colonization in health and disease.

### 7.1 Molecular Assays

Culture‐dependent methods fall short in accurately assessing the true diversity of the intestinal microbiota because a large fraction of the microbiota cannot be cultured [46]. Recent human studies utilizing novel high‐throughput technologies have revealed a previously unseen complexity in the human gut microbiota, with hundreds of phylotypes identified and confirming that up to about 80% remain uncultured [47].

For decades, SIBO was defined as a type of small intestinal dysbiosis, characterized principally by excessive bacterial load in the small intestine [48]. A novel culture‐independent approach for diagnosing SIBO uses a real‐time quantitative polymerase chain reaction (qPCR) based approach targeting the bacterial 16S rRNA gene and the human beta‐actin gene to assess and with optimized deoxyribonucleic acid (DNA) extraction efficiency for recovering the mucosa‐associated microbiome [49]. Subsequently, this approach was applied to objectively quantify the bacterial load on the mucosal biopsies obtained aseptically from the proximal duodenum [7]. A pilot study using this method found that duodenal bacterial load in patients with functional dyspepsia (FD) was inversely related to quality of life and positively related to symptom severity after a nutrient challenge test (a surrogate test for assessing the visceral sensory function) [50].

Beyond having higher duodenal bacterial loads, patients with FD exhibited a greater relative abundance of *Streptococcus* and reduced relative abundances of *Prevotella, Veillonella*, and *Actinomyces* compared with controls, suggesting that their symptoms may stem from microbiome alterations in the proximal duodenum. These findings were later replicated in larger cohorts of patients with gastrointestinal disorders, such as DGBI and inflammatory bowel disease, compared to healthy asymptomatic controls [51, 52]. Furthermore, a recent study [53] showed an inverse link between gastric emptying and relative abundances of *Streptococcus* and *Prevotella*, and the relative abundance of *Veillonella* spp. in the duodenal MAM in patients with FD, while habitual diet and duodenal MAM profiles showed no such associations.

Using 16S rRNA sequencing of duodenal aspirates, subjects with SIBO (diagnosed with a threshold > 10^3^ cfu/mL) had a significantly higher relative abundance of the phylum Proteobacteria (4.31‐fold) and a reduced relative abundance of Firmicutes (1.64‐fold) compared to subjects without SIBO [54]. They also identified a distinct Proteobacterial profile that correlated with symptom severity. A pilot study showed that the MAM exhibits substantial heterogeneity in both the relative and absolute abundances of bacterial profiles across patients with inflammatory bowel disease and controls, and across different anatomical regions of the upper and lower gastrointestinal tract [55]. Total and taxon‐specific absolute microbial loads determined by digital PCR methodology in 250 duodenal aspirates from patients with various gastrointestinal disorders tended to be higher in those with culture‐defined SIBO compared to those without SIBO [56]. Furthermore, enrichment of what they described as “disruptor taxa” (*Enterobacteriaceae*) was found in many SIBO cases, with higher disruptor loads linked to more severe symptoms. This study suggested that the SIBO diagnosis should focus on quantifying a specific group of disruptor taxa rather than on the total microbial load.

A pilot study using 16S rRNA and shotgun sequencing of duodenal aspirates found that SIBO (diagnosed with a threshold ≥ 10^3^ CFU/mL) is associated with higher Escherichia/Shigella and Klebsiella, lower microbial diversity and network connectivity, and altered metabolic pathways that correlate with gastrointestinal symptoms [44]. The data support defining SIBO at a threshold of ≥ 10^3^ CFU/mL and identified *E. coli* and *Klebsiella* strains as primary drivers of overgrowth.

These studies show that measuring microbial load, diversity, and especially, microbial metabolic activity is essential to accurately assess the role of microbes colonizing the MAM in health and disease.

### 7.2 Volatiles Produced by Gastrointestinal Microbes

Microorganisms can also release a plethora of volatiles, and it appears that microbial volatile organic compounds (mVOCs) can play an important role in shaping microbial communities [57]. mVOCs act as long‐range signals influencing the growth of other microbes, while soluble microbial products drive short‐range interactions. mVOCs such as hydrogen sulfide, ammonia, trimethylamine, nitric oxide, and 2‐amino‐acetophenone can modify biofilm formation, dispersal and bacterial motility [58]. The role of mVOCs in human disease is not well studied, but analyzing mVOCs from the gut lumen or biologic samples cultured from the gut could reveal key microbe–microbe interactions in the human gastrointestinal tract.

### 7.3 Luminal Sensing of Microbial Products During Gastrointestinal Transit Utilizing Novel Capsule Technology

Electronic‐based, swallowable capsules that can safely quantify pH or intraluminal gas concentrations in real time and wirelessly transmit data during transit through the gastrointestinal tract have been developed [59]. One of these, the Atmo Capsule, is able to measure concentrations of intraluminal gases, such as hydrogen and carbon dioxide, produced by the metabolic activity of the gut microbiota [60, 61]. Studies to date have demonstrated its potential utility in the colon in defining patterns of fermentation of carbohydrates and protein and their modulation with dietary fiber [61, 62]. Such a capsule may have utility in defining microbial metabolic profiles in the small intestine and hence be useful in the diagnosis and/or characterization of suspected SIBO. Furthermore, the at present unrealized potential to incorporate microsensors that broaden the analytes to include important mVOCs may expand the usefulness of the data yielded; for example, quantification of methane and hydrogen sulfide may enable validation (or otherwise) of the concepts that IMO and ISO have relevance to the small intestine. Beyond being non‐invasive and convenient, such capsules offer advantages over conventional breath tests, which face interpretive challenges associated with not knowing the regional origin of the gas production (as discussed earlier) and from the very low signal‐to‐noise ratio of the output (gases are only measured in parts‐per‐million concentrations).

### 7.4 Research Gaps

Clinicians face a significant challenge in diagnosing small intestinal dysbiosis, more simplistically known as SIBO, due to the limited sensitivity and specificity of clinically available diagnostic tests. This leaves the diagnosis to chance, akin to flipping a coin. Treatments are often empirical without evidence of small intestinal‐specific effects, offering only temporary relief with frequent relapses and requiring a trial‐and‐error approach. This dilemma surrounding diagnosis and treatment contributes to dissatisfaction and frustration among patients and clinicians, fueling a cycle of repeated visits, extensive testing, and ineffective therapies that offer little relief and ultimately prolong suffering.

New tests for characterizing small‐intestine microbial colonization and function are rapidly evolving and aim to meet new standards for reliability, universal validity, and relevance to key gastrointestinal conditions while capturing meaningful patient outcomes (Table 2). They are not yet validated, not ready for routine clinical use, and remain largely confined to research settings. In the future, these novel approaches hold promise for personalized medicine, enabling individualized diagnostics and targeted therapies, eliminating ineffective trial‐and‐error “one‐size‐fits‐all” approaches to the structure and function of the small bowel microbiota.

**TABLE 2:** Science and evidence in the context of small intestinal bacterial overgrowth (SIBO).

Dimension	What it means	SIBO‐specific considerations	Examples
Validity of available data	A measure for the degree to which data reflect true clinical phenomena, not measurement artifacts.	Diagnostic criteria (breath testing vs. culture), outcome definitions, and study designs must capture the real biology of SIBO.	Validated breath tests, standardized culture methods, and consistent diagnostic thresholds.
Reliability (consistency)	Reproducible results across populations, laboratories, and methods.	Challenge in SIBO due to heterogeneous testing protocols, patient phenotypes, and lab variability.	Cross‐lab reproducibility studies, multicenter cohorts, replicated trial findings.
Relevance	Evidence needs to directly address the clinical questions and patient‐centered outcomes.	Endpoints should capture core SIBO outcomes (symptom relief, nutritional status, recurrence prevention, quality of life).	Trials and guidelines focusing on symptoms, bloating reduction, weight changes, and symptom scales relevant to patients.
Level of evidence (hierarchy)	Ranking of evidence strength from highest to lowest.	SIBO research spans SRMAs, RCTs, cohorts, case–control studies, with expert opinion lowest when higher‐quality data are lacking.	Impact supported by systematic reviews/meta‐analyses of randomized controlled trials, prospective cohorts, case–control studies, expert opinion.
Statistical and clinical significance	Differentiation between statistical significance and clinical (real‐world), meaningful benefit.	A small *p*‐value must mirror a relevant improvement in patient outcomes; consistency and effect sizes matter.	Reported effect sizes, confidence intervals, number needed to treat, minimal clinically important differences.

## 8 Conclusions

While the application of breath hydrogen testing to recognize SIBO in a research setting has provided insights into intestinal pathophysiology and disease pathogenesis, its accuracy is insufficient to confidently identify SIBO in an individual patient in the clinical setting, to guide management and to assess efficacy of therapy. However, this does not mean the concept of SIBO is invalid. At worst, it is the testing that feeds a “fake” narrative that further deteriorates with overinterpretation of the breath hydrogen and symptomatic responses to therapy directed towards SIBO. The way forward is to put such imprecision aside, to perform studies in which the level of sophistication and rigor of microbiological analysis is enhanced, and to attempt to prove the associations of small intestinal bacterial populations with respect to their density and/or structure with pathology and symptom generation, and to prove whether their manipulation has specific benefits in patients.

## Funding

This work was supported by the National Health and Medical Research Council (APP2043411 and APP2035319).

## Conflicts of Interest

A.S.: Atmo Biosciences provided support for an investigator‐initiated research project. G.H.: Atmo Biosciences provided support for an investigator‐initiated research project. P.R.G.: Has consulted for and received support for investigator‐driven studies from Atmo Biosciences. Shareholder in Atmo Biosciences. His Department financially benefits from the sales of a digital application (Monash University FODMAP diet app), patient booklets, cookbooks, and online courses all of which relate to the low FODMAP diet therapy. These commercial activities contribute to the salary of P.R.G.

## Data Availability

Data sharing not applicable to this article as no datasets were generated or analysed during the current study.
